# Precision analysis of a whole blood T cell subset immunophenotyping assay

**DOI:** 10.1186/2051-1426-1-S1-P96

**Published:** 2013-11-07

**Authors:** Minjun Apodaca, Stephen C De Rosa, Leonard A D'Amico, Bryan J Kennedy, Mary L Disis, Chihiro Morishima

**Affiliations:** 1 Medicine, University of Washington, Seattle, WA, USA; 2 Laboratory Medicine, University of Washington, Seattle, WA, USA

## Background

The Cancer Immunotherapy Trials Network has initiated several clinical trials utilizing T cell growth factors such as IL-15 and IL-7. In support of these trials, we have developed and conducted precision testing of a 12-color flow cytometric assay to characterize the surface markers present on T cells in fresh whole blood and among cryopreserved peripheral blood mononuclear cells (PBMC).

## Methods

A combination of 12 antibodies specific for the following T cell surface markers was evaluated using a Becton Dickinson (BD) LSRII flow cytometer: anti-CD45, anti-CD3, anti-CD4, anti-CD8, anti-CD45RA, anti-CD197 (CCR7), anti-CD28, anti-CD25, anti-CD127 (IL-7 receptor), anti-HLA-DR, anti-CD279 (PD-1), and anti-CD278 (ICOS). Whole blood samples were incubated with a cocktail of antibodies, treated with BD Pharm Lyse, fixed with 4% paraformaldehyde, and frozen in 10% DMSO at -80°C. PBMC samples were treated similarly except that they were fixed with 1% paraformaldehyde and kept at 4°C. Samples were run on a flow cytometer within 2 days and analyzed using FlowJo software. Whole blood samples were thawed prior to running on the flow cytometer. Percentages of T cell subsets were assessed for within-assay and inter-operator variation from the whole blood of 3 healthy volunteers, while within-assay and inter-assay variation (across multiple days) was assessed using PBMC from 1 healthy volunteer.

## Results

For stable markers that define T cell memory subsets, including CD4, CD8, CD45RA, CD197, and CD28, the within-assay coefficient of variation (%CV) in whole blood ranged from 0-7.2% for the 3 different donors, among 3 unique operators (mean range of marker expression: 20.4-99.9%). The inter-operator CV for these markers ranged from 0.1-5.7% in whole blood for the 3 different donors and among 3 operators. As expected, measures of T cell activation such as CD25, HLA-DR, PD-1 and ICOS were expressed at substantially lower levels in these samples from healthy volunteers (mean range of expression: 0.3-33.8%), and the %CVs were correspondingly higher (0.6%-100.8%). Among cryopreserved PBMC, within-assay CVs for T cell memory subset markers ranged from 0-18.6%, while inter-assay variation measured over 3 assay days ranged from 0.3-13.7%.

## Conclusions

We have designed a flow cytometric staining panel that permits the identification of memory T cell subsets as well as activated T cells in whole blood. The assay precision for memory T cell surface markers was excellent at <7.3%, which will allow the identification of modest changes in the percentage of memory populations over time. Proportionally larger differences will be required to precisely ascertain changes in the percentages of activated T cells.

